# Cell-retention for lipid production in continuous *Schizochytrium limacinum* SR21 cultures fed with dark fermentation effluent

**DOI:** 10.1186/s40643-026-01120-6

**Published:** 2026-08-31

**Authors:** Simon Täuber, Leonie Ackermann, Henry Schittkowski, Peter Neubauer, Stefan Junne

**Affiliations:** 1 Bioprocess Engineering, Department of Biotechnology, Technische Universität Berlin, Ackerstraße 76 ACK24, 13355 Berlin, Germany; 2 Department of Chemistry and Bioscience, Aalborg University Esbjerg, Niels Bohrs Vej 8, 6700 Esbjerg, Denmark

**Keywords:** Volatile fatty acids, Microbial hydrolysis, Docosahexaenoic acid, Polyunsaturated fatty acids, *Aurantiochytrium limacinum*, Membrane bioreactor

## Abstract

The marine protist *Schizochytrium limacinum* SR21 is a promising producer of docosahexaenoic acid (DHA) from short-chain carboxylic acids (SCCAs). Dark fermentation effluent (DFE) is an SCCA- and nutrient-rich substrate derived from biogenic residues and has previously been evaluated for microbial lipid production. However, its low carbon concentration limits volumetric productivity and causes substantial culture dilution when larger feed volumes are required.

Membrane-based cell retention is an established process intensification strategy for low-concentration feed solutions, as it decouples hydraulic residence time from biomass residence time. However, the combination of DFE utilisation and cell retention for DHA production by *S. limacinum* has received little attention. In this study, repeated fed-batch and cell retention strategies were evaluated to overcome dilution limitations and enable intensified biomass and lipid production from DFE. Additional cultivations with defined SCCA mixtures showed preferential uptake of acetate and butyrate over propionate and lactate, which was also observed during DFE conversion. In repeated fed-batch operation, biomass output reached 7.5 g after 95 h. In contrast, a final biomass output of 33 g was achieved in cell retention mode, representing a 4.4-fold increase. This improvement was not associated with enhanced substrate conversion efficiency but resulted from retaining biomass while increasing DFE throughput. While applying cell retention, the specific palmitic acid concentration increased to 116 mg g⁻¹, while DHA remained comparatively stable at 57 mg g⁻¹. This resulted in a DHA titre of 1.9 g L⁻¹ compared with 0.24 g L⁻¹ in repeated fed-batch. Nitrogen and phosphate removal reached 58% and 87%, respectively. However, the relatively high nutrient content of DFE limited the establishment of strong nitrogen limitation.

Overall, cell retention enabled process intensification with a total lipid space-time yield of 0.07 g_lipids_ L⁻¹ h⁻¹ and a DHA space-time yield of 19.7 mg L⁻¹ h⁻¹. The results demonstrate that cell retention is an effective strategy for valorising low-concentration DFE while operational boundaries for DFE-based DHA production are identified.

**Graphical abstract:**

**Figure:**
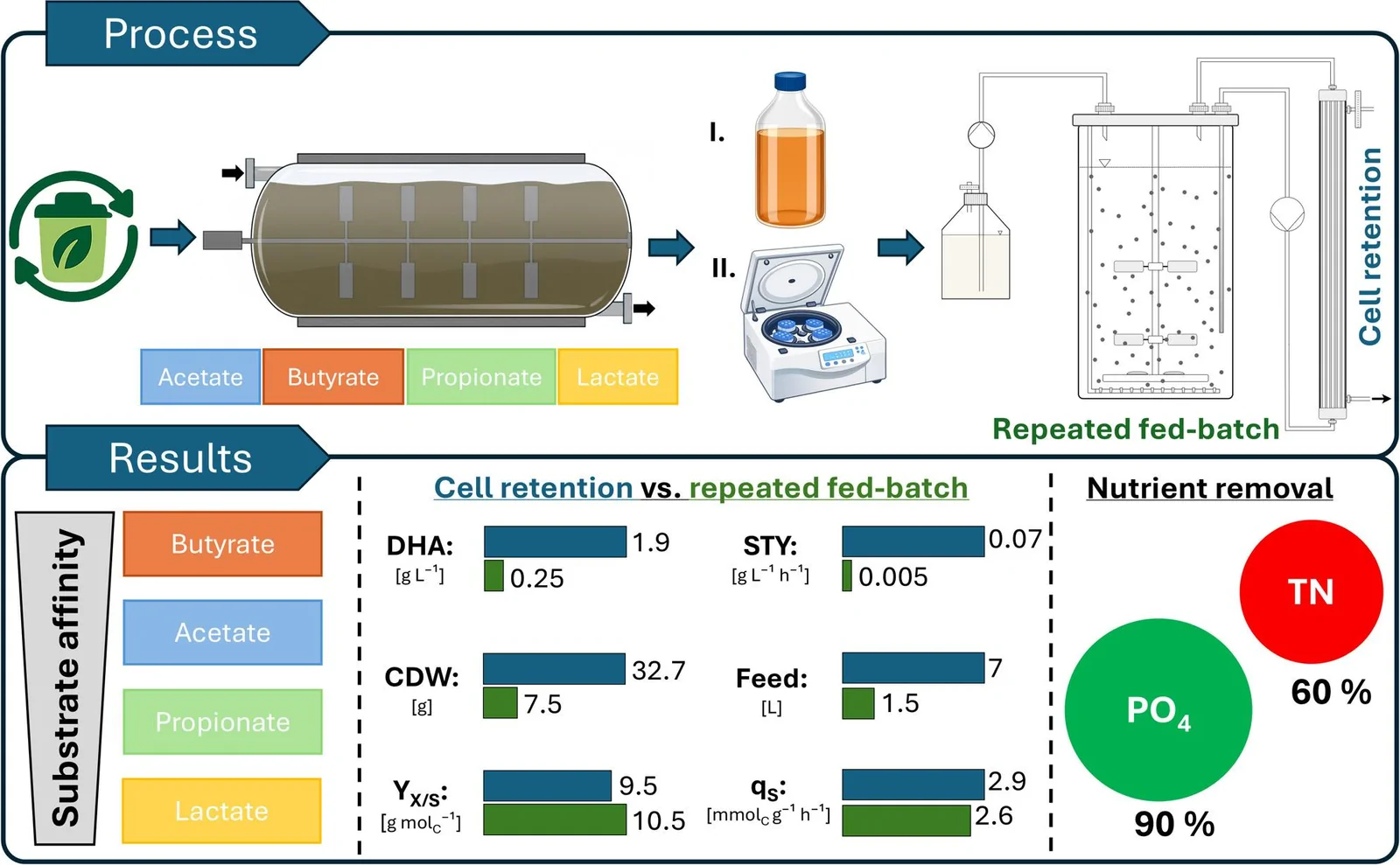

## Highlights

*Schizochytrium limacinum* SR21 effectively utilises dark fermentation effluent for lipid production.

Cell retention increases cumulative biomass output 4.4-fold compared with repeated fed-batch operation.

Cell retention achieves a DHA titre of 1.9 g L⁻¹ and a DHA yield of 510 mg mol_C_⁻¹ during DFE conversion.

DFE conversion removes 58% of total nitrogen and 87% of phosphate.

A substrate preference hierarchy from acetate and butyrate (high) to propionate and lactate (low) is identified.

## Introduction

*Schizochytrium limacinum* SR21, also known as *Aurantiochytrium limacinum*, is a widely applied producer of polyunsaturated fatty acids such as docosahexaenoic acid (DHA) (Tocher et al. 2019). DHA is essential for human nutrition (Djuricic and Calder 2021) and is used as a supplement in aquaculture fish feed (Naylor et al. 2021). Although fish are a valuable dietary source of DHA, they do not synthesize it in meaningful quantities. Instead, DHA enters the food web through microbial and algal producers (Tocher 2010). To reach DHA concentrations comparable to those in wild fish, aquaculture fish require supplementation with omega-3-rich ingredients. These are commonly supplied through fish meal and fish oil. This practice is seen as critical, as the consumption of fish meal and oil puts pressure on marine ecosystems due to overfishing (Bartek et al. 2021; Naylor et al. 2021). Microbial DHA-rich oils offer a more resource-efficient alternative. Recent studies show that lipids from *S. limacinum* can replace fish oil in feeds for Nile Tilapia (Sarker et al. 2016) and salmon (Katerina et al. 2020) without compromising growth or product quality.

A further step toward sustainability is the use of second-generation feedstocks. Converting biogenic residues into microbial lipids reduces the competition with carbon substrates for food use. Dark fermentation (DF) can convert biogenic residues into SCCA-rich liquid substrates. In DF, mixed microbial consortia degrade carbohydrates and other biodegradable fractions under anaerobic, non-methanogenic conditions, producing an acidogenic broth that is rich in SCCAs such as acetate, butyrate, propionate, and sometimes lactate (Menzel et al. 2020). The process is robust and can be operated with diverse feedstocks, which makes it particularly attractive for heterogeneous waste streams. The SCCA mixture, often referred to as the carboxylate platform, provides a versatile intermediate that can be redirected toward value-added bioprocesses instead of energy recovery alone (Chalima et al. 2021; Täuber et al. 2025b). Currently, DF is mainly applied as a microbial hydrolysis step as part of a full anaerobic digestion to improve methane yields and enhance the digestibility of recalcitrant biomass (Janesch et al. 2021). In this study, the acidogenic liquid phase was used as feedstock for microbial lipid synthesis.

SCCA-based lipid production has been demonstrated across yeasts (Krikigianni et al. 2022), microalgae (Patel et al. 2022), and marine protists (Chalima et al. 2021; Shafiq et al. 2024; Zhang et al. 2025) with both pure acids and DFE. As mentioned earlier, one of the main challenges for the application is the low carbon content of DFE, which constrains volumetric performance and results in low concentration of biomass and lipid titres in conventional operation due to dilution (Täuber et al. 2025a).

Cell retention offers a direct process engineering strategy to address this limitation. By retaining biomass in the reactor while allowing cell-free permeate removal, hydraulic throughput can be increased without washing out the production organism. Hollow fibre membrane modules are widely used for this purpose as they provide high membrane areas in compact and scalable configurations. Current membrane-based cell retention systems differ mainly in membrane material, pore size, fouling control, and process monitoring. Polyether sulfone is frequently used in laboratory-scale hollow fibre modules because it combines chemical robustness, compatibility with alkaline cleaning, and practical handling. Alternative materials such as polyvinylidene fluoride and ceramics can provide higher chemical or mechanical stability, but are often associated with higher cost or lower flexibility in early-stage process development (Asif and Zhang 2021). For microbial cell retention, pore sizes of 0.2 to 0.45 μm are commonly applied in processes with bacteria (Burniol-Figols et al. 2020; Schroedter et al. 2025) and yeasts (Paul et al. 2019).

Recent developments also focus on improving long-term membrane operation. Hybrid and composite membranes with antifouling coatings or self-cleaning properties have been investigated to reduce fouling (Wang et al. 2023). Operational strategies such as periodic backflushing, pulsed flow, and alternating flow directions can further support stable permeate flux, especially when complex or raw substrates are processed as shown by Corsino et al. (2022) and Wang et al. (2021). In addition, transmembrane pressure sensors, online turbidity measurements, and flow control can improve process reliability by enabling fouling detection and automated cleaning cycles as discussed by Akkoyunlu et al. (2021). Hollow fibre membrane (HFM) based cell retention has already improved productivity in lactic acid production (Schroedter et al. 2025), polyhydroxyalkanoate production (Burniol-Figols et al. 2020), and lipid production with the oleaginous yeast *Cutaneotrichosporon oleaginosus* (Koruyucu et al. 2023). However, its application to DFE-based DHA production with *S. limacinum* remains largely unexplored.

Since waste-derived feeds seldom have a fixed composition, the relative concentrations of acetate, butyrate, propionate, and lactate fluctuate over time (Menzel et al. 2020). Each acid affects growth and lipid metabolism differently, and therefore, the final product profile (Chalima et al. 2021; Shafiq et al. 2024; Zhang et al. 2025). Prior work with *S. limacinum* SR21 points to a preference for acetate and butyrate, whereas propionate tends to support lower productivities (Chalima et al. 2020; Patel et al. 2021; Zhang et al. 2025). However, data quantifying diauxic transitions, especially with DFE as feed, remain scarce.

DFE also contains macronutrients such as nitrogen and phosphate. Their availability creates a process trade-off. On the one hand, nitrogen and phosphate support biomass formation and can be assimilated into microbial biomass. On the other hand, high nutrient availability can delay or prevent the nutrient limitation typically required for strong lipid accumulation in oleaginous microorganisms (Junne et al. 2021). Therefore, the C/N/P ratio and the bioavailability of nitrogen and phosphate are important for both process performance and product formation.

DFE may contain inhibitory compounds: (i) furan derivatives, such as furfural and hydroxymethylfurfural, that arise from the dehydration of pentoses and hexoses, and (ii) phenolic compounds that are released during lignin depolymerisation under acidic or thermal conditions (Monlau et al. 2014; Lyu et al. 2025). These compounds are known to interfere with cellular membranes, central metabolic pathways, and overall growth performance in many microorganisms, including oleaginous species (Lyu et al. 2025). Therefore, potential inhibitory effects should be considered at the process level when evaluating DFE as a feedstock for microbial lipid production.

**Fig. 1:**
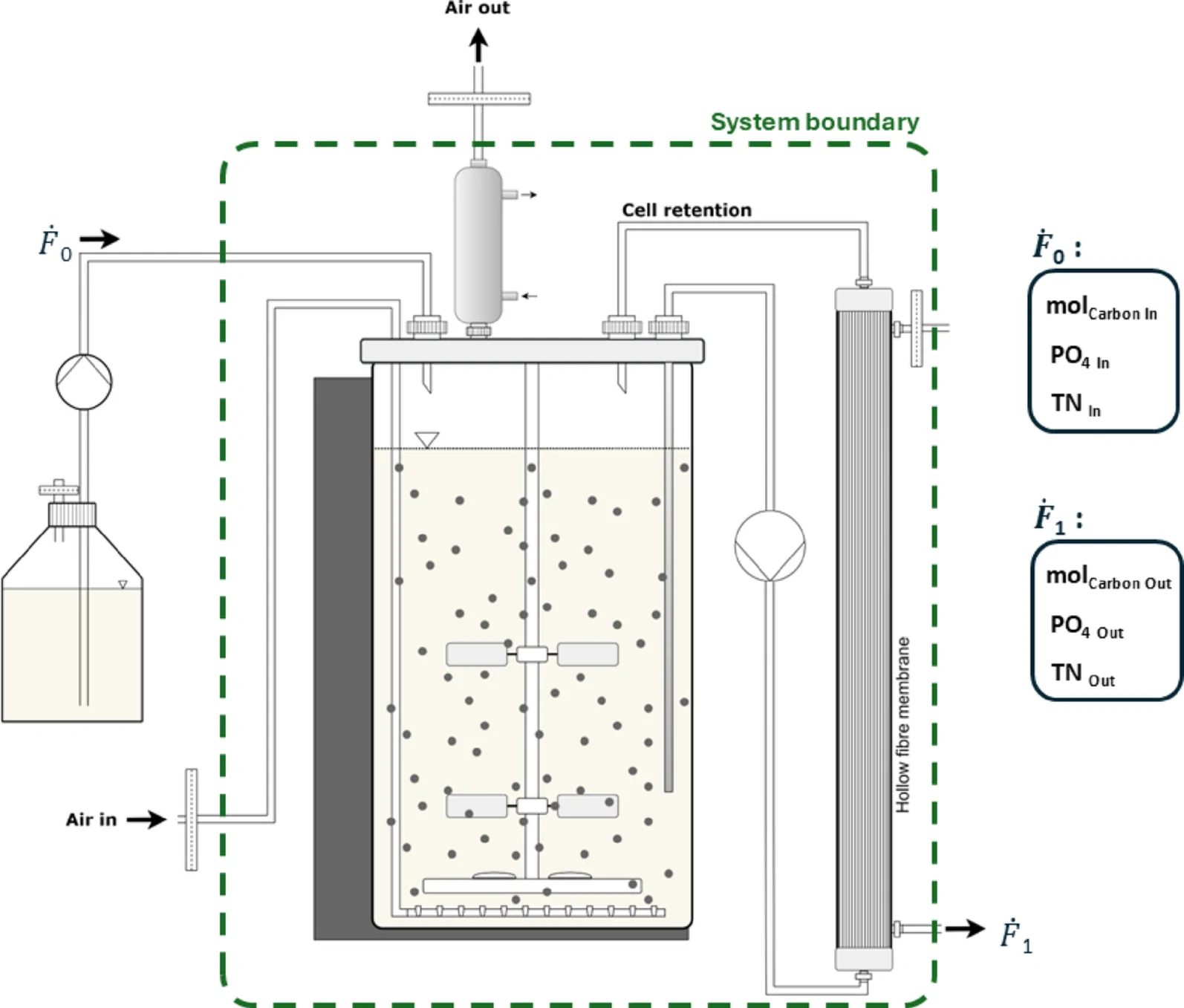
Schematic representation of the bioreactor system with external cell retention. The system boundary (green dashed line) includes a 1 L stirred-tank reactor and a hollow fibre membrane module. Feed was supplied via pH control (Ḟ₀), and spent medium was withdrawn as cell-free permeate (Ḟ₁), carrying the corresponding output of carbon, phosphate, and nitrogen

This study aimed to demonstrate lipid production from DFE with a focus on DHA production and to test whether cell retention enables higher titres and productivity by overcoming volumetric limits. We compared a repeated fed-batch (RFB) strategy with a cell retention cultivation mode (CR), the latter with a hollow fibre membrane loop (Fig. 1). In addition, we quantified substrate uptake in a defined mixture of acetate, butyrate, propionate, and lactate and compared these patterns with DFE conversion to assess substrate preferences. Finally, we monitored nitrogen and phosphate and examined C/N/P ratios to assess the potential to couple nutrient removal with lipid production.

## Materials and methods

### Dark fermentation effluent

The DFE used in this study was generated in a laboratory-scale plug-flow reactor as described by Menzel et al. (2023a). The reactor had a maximum working volume of 14 L and was operated under mesophilic conditions at a stirring speed of 5 rpm and a hydraulic retention time of 14 days.

The fermentation was conducted over several months and included phases of bioaugmentation with *Paenibacillus glucanolyticus* and *Paenibacillus macerans*, which increased SCCA concentrations to a maximum of 10.3 g L^-1^ butyrate and 6 g L^-1^ acetate (Menzel et al. 2023a). The reactor displayed typical temporal variability in SCCA profiles, a characteristic of long-term acidogenic operation under both native and augmented microbial communities. For this study, DFE was collected continuously over several weeks and blended, resulting in three compositions (DFE1 to DFE3, Table 1). DFE was collected and filtered through a 0.125 mm sieve before being frozen at -20 °C. Before use, DFE was thawed, centrifuged at 1,610 x g for 10 min, and autoclaved.

**Table 1:** Composition of three dark fermentation effluents as applied in this study

Parameter	DFE 1	DFE 2	DFE 3
Acetate	4.16 ± 0.17	3.79 ± 0.01	5.03 ± 0.07
Butyrate	9.75 ± 0.21	7.39 ± 0.02	6.26 ± 0.12
Propionate	0.60 ± 0.01	0.81 ± 0.02	1.54 ± 0.05
Lactate	0.25 ± 0.01	0.37 ± 0.01	0.14 ± 0.01
COD	26.8 ± 3.3	23.4 ± 3.5	23.5 ± 6.1
TN	204 ± 15	229 ± 17	222 ± 23
PO_4_	261 ± 13	255 ± 16	264 ± 2
Molar C	0.62	0.52	0.55
C/N/P	42/1/0.19	32/1/0.16	35/1/0.18
pH	3.9	4.0	4.0
Cultivation	CR1	CR2, CR3	RFB

Values represent mean ± standard deviation of three independent measurements of short-chain carboxylic acids (g L^-1^), chemical oxygen demand (COD) (g L^-1^), total nitrogen (TN) (mg L^-1^), phosphate (PO₄) (mg L^-1^), and molar carbon content (mol_Carbon_ L⁻¹). Molar C/N/P ratios were calculated from SCCAs, total nitrogen, and phosphorus concentrations. Corresponding experiments are listed in the last row

### Strain, medium composition, and seed train

*Schizochytrium limacinum* SR21, also known as *Aurantiochytrium limacinum* SR21, was obtained from the American Type Culture Collection (ATCC® MYA 1381™) and used throughout all cultivations. Cryogenic stocks were stored at − 80 °C in 20% w w⁻¹ glycerol.

The cultivation medium contained the following components in g L⁻¹: Na₂SO₄ 10.0, MgCl₂ · 6 H₂O 2.44, CaCl₂ · 2 H₂O 0.30, KCl 0.6, NaNO₃ 1.0, NH₄Cl 0.03, KH₂PO₄ 0.05, glucose 10.0, yeast extract 1.0, peptone 1.0, thiamine 0.01, biotin 5 × 10⁻⁴, and cyanocobalamin 5 × 10⁻⁴. A trace element solution was added at 5 mL L⁻¹ and contained, in g L⁻¹: Na₂EDTA 6.0, FeCl₃ · 6 H₂O 0.3, MnCl₂ · 4 H₂O 0.86, ZnCl₂ 0.06, CoCl₂ · 6 H₂O 0.03, CuSO₄ · 5 H₂O 0.002, H₃BO₃ 1.0, NiCl₂ · 6 H₂O 0.05, and Na₂MoO₄ · 2 H₂O 0.005.

Na₂SO₄ was used as the main sodium salt instead of NaCl to provide a saline medium while reducing chloride-induced corrosion (Hu et al. 2015). The initial medium pH was adjusted to 6.5 with 1 M H_2_SO_4_ before sterilization. Glucose was sterilized separately, and heat-sensitive components, including vitamin solution and trace element solution, were sterilized by filtration through 0.2 μm filters.

Initial cultures from cryostocks were grown on agar plates containing the cultivation medium supplemented with 20 g L⁻¹ agar for three days at 30 °C. Cells were then transferred to 250 mL Ultra Yield® flasks containing 50 mL preculture medium, consisting of the cultivation medium with a total of 20 g L⁻¹ glucose, and cultivated for 48 h at 30 °C and 200 rpm on an orbital shaker with an amplitude of 50 mm. A second preculture was performed in 1.5 L shake flasks containing 200 mL of preculture medium for another 48 h under the same incubation conditions. The second preculture was used to inoculate all bioreactor experiments with 5% (v v⁻¹) inoculum.

### Bioreactor operation

Bioreactor cultivations were conducted in stirred bench-scale bioreactors (MultiFors, Infors HT, Switzerland) operated at 1 L working volume. Bioreactors were sterilized by autoclaving at 121 °C for 20 min. During cultivation, temperature was maintained at 30 °C and the pH at 6.5 by addition of either DFE or 1 M sulfuric acid. Dissolved oxygen was kept above 35% by adjusting the agitation frequency between 150 and 800 rpm and the aeration rate between 0.25 and 1 vvm. Dissolved oxygen, pH, stirring speed, temperature, and off-gas composition were monitored *online*. Feed addition was recorded gravimetrically. Culture volume was controlled according to the respective cultivation mode described below.

### Defined SCCA mixture cultivations

Defined SCCA mixture cultivations were performed in bioreactors to quantify the SCCA uptake behaviour of *S. limacinum* SR21 under defined process conditions. Glucose in the culture medium was replaced by a defined SCCA mixture. The initial medium contained, in g L⁻¹, acetate 5.0, butyrate 5.0, propionate 2.5, and lactate 1.0. These acids were supplied as their respective sodium salts.

After depletion of the initially supplied acetate and butyrate and after the onset of reduced growth, a second SCCA pulse was added. For this pulse, the corresponding sodium salts of acetate, butyrate, propionate, and lactate were dissolved in 50 mL of sterile water and added directly to the bioreactor to reach target concentrations of 5.0 g L⁻¹ acetate, 5.0 g L⁻¹ butyrate, 2.5 g L⁻¹ propionate, and 1.0 g L⁻¹ lactate. The pH was maintained at 6.5 throughout cultivation by addition of 1 M sulfuric acid.

### Repeated fed-batch cultivation with DFE

Repeated fed-batch cultivation was used as a non-membrane-based reference strategy for DFE application. The process started with an initial batch phase at 50% filling and containing 10 g L⁻¹ glucose. After glucose was almost depleted, DFE feeding started in pH-auxostat mode at pH 6.5. In this setup, cellular consumption of SCCAs caused the culture pH to increase, which triggered the addition of DFE. As soon as 1 L of reactor filling was reached, 50% of the reactor broth was removed and replaced by DFE addition through pH-controlled feeding (here at approx. 45 and 62 h). The removed broth contained both cells and spent medium. The cumulative DFE feed volume and cumulative culture withdrawal volume were recorded gravimetrically. Two repeated fed-batch cultivations were performed and are further reported as biological process replicates.

### Cell retention cultivation with DFE

Cell retention cultivations were performed to increase the throughput of dilute DFE while retaining biomass within the reactor. The bioreactor was configured as described for the RFB cultivations and connected to an external hollow fibre membrane module through a recirculation loop. The Midikros® module consisted of polyethersulfone and had a pore size of 0.2 μm and a membrane area of 180 cm². Culture broth was circulated through the membrane module at a mean crossflow rate of 7.80 L h⁻¹. The retentate was returned to the reactor, while cell free permeate was removed.

Permeate withdrawal was performed intermittently and adjusted to the pH-controlled DFE feed rate. Whenever the reactor volume reached approximately 1.1 L, the membrane loop was activated until 0.2 L of permeate had been removed, reducing the reactor volume to 0.9 L. Therefore, each withdrawal corresponded to approximately 20% of the nominal working volume.

The average hydraulic retention time (HRT) during the DFE feeding phase was calculated from the mean reactor volume, feeding duration, and cumulative feed volume. This resulted in an average HRT of 10.4 h. Since no deliberate biomass withdrawal occurred and the permeate was cell-free, the biomass retention time equals the process time. The dilution rate was calculated by dividing the feed rate F by the filling volume V, where F is the feed rate of the DFE. The recirculation flow through the membrane module was excluded from this calculation as the retentate was returned to the reactor. Maximum DFE feed flow rates were 156, 120, and 140 mL h⁻¹ for CR1, CR2, and CR3, respectively.

Three cell retention cultivations were performed and are denoted as CR1, CR2, and CR3. These runs were conducted with different DFE batches that varied slightly in SCCA, nitrogen, and phosphate composition, reflecting the inherent variability of DFE from mixed microbial acidogenic fermentation. Therefore, CR1 to CR3 are shown both individually and in a comparison to repeated fed-batch operation at process level.

To reduce membrane fouling, the hollow fibre membrane module was disconnected after 48 h of DFE feeding and backflushed for 10 min with 1 M NaOH, followed by 10 min flushing with sterile water. The transmembrane pressure was not monitored in this study. Membrane performance was therefore assessed operationally based on the ability to maintain permeate withdrawal and reactor volume control. During 95 h of cultivation, it was possible to maintain the permeate flow to keep the reactor volume within the predefined range without additional efforts.

### Analytical methods

#### Total nitrogen

Total nitrogen was quantified with a LCK338 photometer kit (Hach Lange). Digestion followed the manufacturer’s protocol using an LT200 thermostat. Absorbance was measured at 345 nm on a DR3900 spectrophotometer (Hach Lange). Measurements were done in duplicate.

#### Nitrate

Nitrate was measured photometrically using Hach LCK339 cuvette tests according to the manufacturer’s protocol. Samples were diluted with deionized water to fit the measurement range. Absorbance was read with a DR3900 spectrophotometer (Hach Lange).

#### Ammonia

Ammonia was measured in the supernatant using the Cedex Bio HT Analyzer with the NH₃ Bio HT test kit according to the manufacturer’s protocol (Roche Diagnostics International AG, Switzerland).

#### Phosphate

Phosphate was determined photometrically using a modified molybdenum blue assay according to Saheki et al. (1985). Filtered samples were acidified with 1 M HCl, mixed with the respective colour reagents, incubated for 60 min at 30 °C, and absorbance was measured at 850 nm with an Ultrospec 2100 Pro (Amersham Bioscience).

### Chemical oxygen demand (COD)

COD was quantified photometrically using Hach LCK314 cuvette tests according to the manufacturer’s protocol. Samples were diluted with deionized water to fit the measurement range. In brief, 2.0 mL of diluted sample was transferred into the cuvette, thermally digested for 2 h at 148 °C in an LT200 thermostat, cooled to room temperature, and measured with the DR3900 spectrophotometer.

### Lipid extraction and quantification

Fatty acids were purified and transesterified (Marbà-Ardébol et al. 2017). The culture broth was diluted to an OD₆₀₀ of 6.0 in 20 mL of deionized water, centrifuged at 1610 x g for 10 min, and washed before resuspension in 500 µL of dry methanol. A 10:1 mixture of methanol and acetyl chloride was prepared, and 2 mL of this mixture was added to the sample. Nonadecanoic acid was used as an internal standard. Samples were esterified at 50 °C for 16 h.

Extraction was conducted with 5 mL of hexane. The upper phase was transferred to a pear-shaped flask. This process was repeated, and the hexane was evaporated using a rotary evaporator at 210 mbar and 50 °C. The fatty acids were redissolved in 1.5 mL of hexane and analysed using a GC with a flame ionization detector (GC-2010, Shimadzu). A dimethylpolysiloxane column (CP Sil 5 CB, Varian) was used at a temperature of 150 °C. The injector and detector temperatures were set to 290 °C and 300 °C, respectively. Gas flow rates were maintained at 30 mL min⁻¹ N₂, 400 mL min⁻¹ synthetic air, and 40 mL min⁻¹ H₂ for the ionization flame.

### Substrate uptake

For all cultivations, substrate uptake was determined for each sampling interval using a mass balance that accounted for substrate addition with feeding DFE, changes in the substrate mass in the reactor’s filling volume, and substrate removal with the permeate. The specific uptake rate was calculated as follows:

1 $$ q_{s} = \frac{{m_{{s,{\mathrm{in}}}} + m_{{s,t_{1} }} - m_{{s,t_{2} }} - m_{{s,{\mathrm{out}}}} }}{{\bar{m}_{X} \Delta t}} $$

  

In Eq. 1, $\:{m}_{s,in}\:$represents the substrate mass inflow, $\:{m}_{s,{t}_{1}}-{m}_{s,{t}_{2}}$ represents the change of the substrate mass within the time time interval, $\:-{m}_{s,out}\:$the substrate mass outflow, and ${\bar{m}_{X} }$the mean biomass present in the bioreactor volume during the time interval. $\:{q}_{s,max}$was determined as the maximum value among time intervals.

### Biomass

To determine cell dry weight, 1 mL of culture broth was centrifuged (21,500 × g, 10 min, 4 °C) in pre-weighed tubes. The supernatant was stored at − 20 °C for further analysis. Cell pellets were washed with deionized water, vortexed, and centrifuged again. The tubes were dried at 80 °C for 48 h and weighed.

### Carbohydrates and short-chain carboxylic acids

The thawed supernatant was centrifuged (21,500 × g, 20 min, 4 °C) and filtered through a 0.2 μm filter before analysis. Analytes were quantified using an HPLC-RID (1200 series, Agilent Technologies) equipped with an H⁺ column (Hyper REZ XP, Thermo Fisher) heated to 65 °C. The eluent (5 mM H₂SO₄) had a flow rate of 0.6 mL min⁻¹. An internal standard (2-butanone) was automatically mixed 1:1 with the sample volume before injection.

## Results

### Diauxic uptake in a defined SCCA mixture

The first objective of this study was to resolve SCCA uptake patterns and potential diauxic shifts in *S. limacinum* SR21. It can metabolise SCCAs to different extents as its sole carbon source. Acetate and butyrate are preferred (Patel et al. 2020), whereas propionate is tolerated but leads to lower growth (Zhang et al. 2025).

**Fig. 2:**
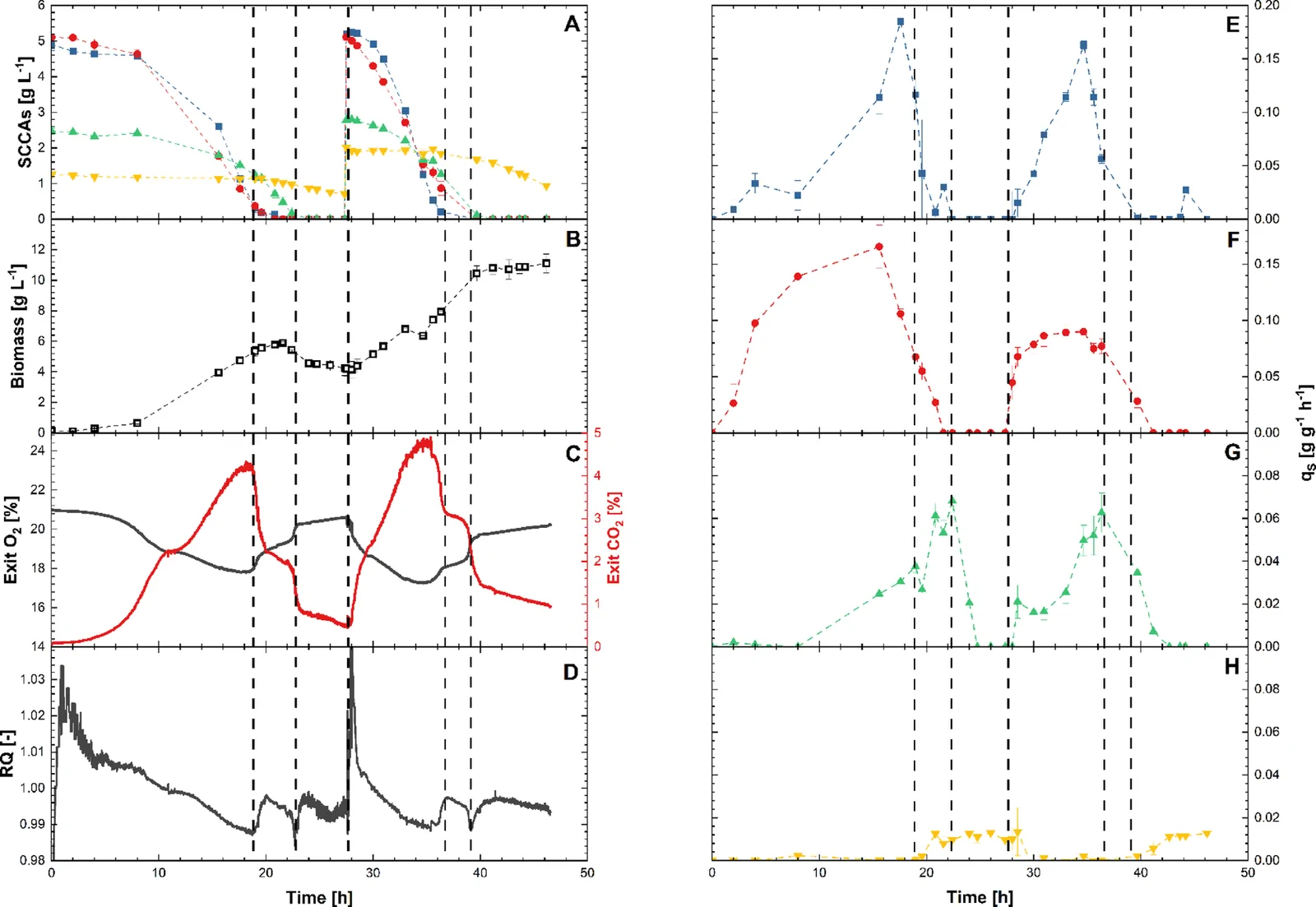
Performance of *S. limacinum* SR21 cultures when using a defined SCCA mix. **A**: Time courses of short-chain carboxylic acids, acetate (blue squares), butyrate (red circles), propionate (green triangles), lactate (yellow inverted triangles); **B**: biomass concentration; **C**: off-gas concentrations of oxygen (black line) and carbon dioxide (red line); **D**: respiratory quotient; **E–H**: specific uptake rate (qₛ) for acetate (**E**), butyrate (**F**), propionate (**G**), and lactate (**H**). Dashed vertical lines indicate distinct transitions in RQ and substrate availability; 3rd line from the left additionally the feed pulse. Values represent the mean of measurements from two independent cultivations, with error bars extending from the lower to the higher value

To investigate this further, *S. limacinum* was cultivated in 1 L bioreactors with a defined SCCA mix as feed. The applied concentrations reflect a plausible DFE composition while not being inhibitory (Shafiq et al. 2024; Zhang et al. 2025). After depletion of the preferred acids and the onset of growth retardation, the same SCCA mixture was added as a second pulse. Figure 2 shows concentration profiles and uptake rates. Acetate and butyrate were consumed first. They were almost depleted within 20 h. Butyrate uptake started immediately and reached a q_s,max_ of 163 mg g⁻¹ h⁻¹ with an overall qₛ of 44 mg g⁻¹ h⁻¹. Acetate uptake was measured later, but with a q_s,max_ of 185 mg g⁻¹ h⁻¹ and a qₛ of 44 mg g⁻¹ h⁻¹. This preference agrees well with previous studies (Shafiq et al. 2024; Zhang et al. 2025).

Propionate uptake was hardly measurable during the first 10 h. The highest rate, 78 mg g⁻¹ h⁻¹, was reached at 22 h, only after butyrate and acetate were depleted. During the second pulse, propionate was taken up in parallel with acetate and butyrate and reached an average qₛ of 23 mg g⁻¹ h⁻¹. The switch to parallel propionate uptake after the second pulse suggests the activation of propionate assimilation during the first fed-batch phase. Hence, simultaneous uptake of the three SCCAs was detected. Propionate was usable but not preferred and can become toxic at concentrations above 5 g L^− 1^ (Zhang et al. 2025). In the mixed SCCA experiment, lactate uptake started only after both acetate and butyrate were depleted. Interestingly, the presence of propionate did not further delay lactate consumption. Lactate reached a q_s,max_ = 13 mg g⁻¹ h⁻¹ and an overall qₛ = 7 mg g⁻¹ h⁻¹. Assimilation proceeds via lactate dehydrogenase to pyruvate, a comparably slow conversion, associated with poor lipid accumulation, as previously discussed (Täuber et al. 2025a). This is relevant for DFE feeding, where lactic acid can be abundant due to lactic acid fermentation. However, suitable DF operation, including HRT and substrate choice, can shift the acid spectrum toward acetate and butyrate and suppress the accumulation of lactate as discussed by Janesch et al. (2021); Menzel et al. (2023b).

While acetate and butyrate were present, biomass increased, with a µ_max_ of 0.280 h⁻¹ during the first phase. Growth slowed during the propionate uptake phase, and biomass declined during the lactate phase. Growth resumed only after the second pulse of acetate and butyrate.

Off-gas analysis supported these interpretations. CO₂ concentration rose during acetate and butyrate catabolism and decreased during propionate and lactate uptake. The respiratory quotient tracked the switches with sharp transitions (dashed lines, Fig. 2) and remained close to 1, which is consistent with oxidative carboxylate metabolism.

### Biomass formation and reactor performance

A major challenge of applying DFE is its low carbon content. Although studies have demonstrated that DFE can serve as a substrate (Patel et al. 2021; Shafiq et al. 2024; Zhang et al. 2025), limitations in working volume and dilution inevitably reduce titre and productivity. To address this, we compared two mitigation strategies for diluted feeds: (i) repeated fed-batch (RFB, *n* = 2) with 50% medium withdrawal per cycle and (ii) cell retention cultivation (CR1–3) with a hollow fibre loop.

For RFB, a biomass concentration of only 4.3 ± 0.1 g L⁻¹ was achieved after 95 h, with an average withdrawal of 0.7 L medium, corresponding to a cumulative biomass of 7.5 ± 0.9 g. In contrast, CR cultivations reached a biomass concentration of 36.2 ± 0.2, 28.9 ± 0.2, and 33.0 ± 0.6 g L⁻¹ after 95 h, yielding an approximately 4.4-fold higher cumulative output. During the DFE feeding phase (24–95 h), specific growth rates were 0.029, 0.026, and 0.035 h⁻¹ for CR1–CR3, with corresponding biomass productivities of 0.44, 0.35, and 0.41 g L⁻¹ h⁻¹. The RFB cultivations achieved 0.031 h⁻¹ and 0.14 g L⁻¹ h⁻¹. Biomass yields were similar across modes: 10.1, 9.5, and 9.0 g_CDW_ mol_C_⁻¹ for CR and 10.5 g_CDW_ mol_C_⁻¹ for RFB cultivations. Comparable Y_x/s_ and µ values for the two modes indicate that the performance gain with cell retention arose from higher medium throughput rather than enhanced cell growth.

**Fig. 3:**
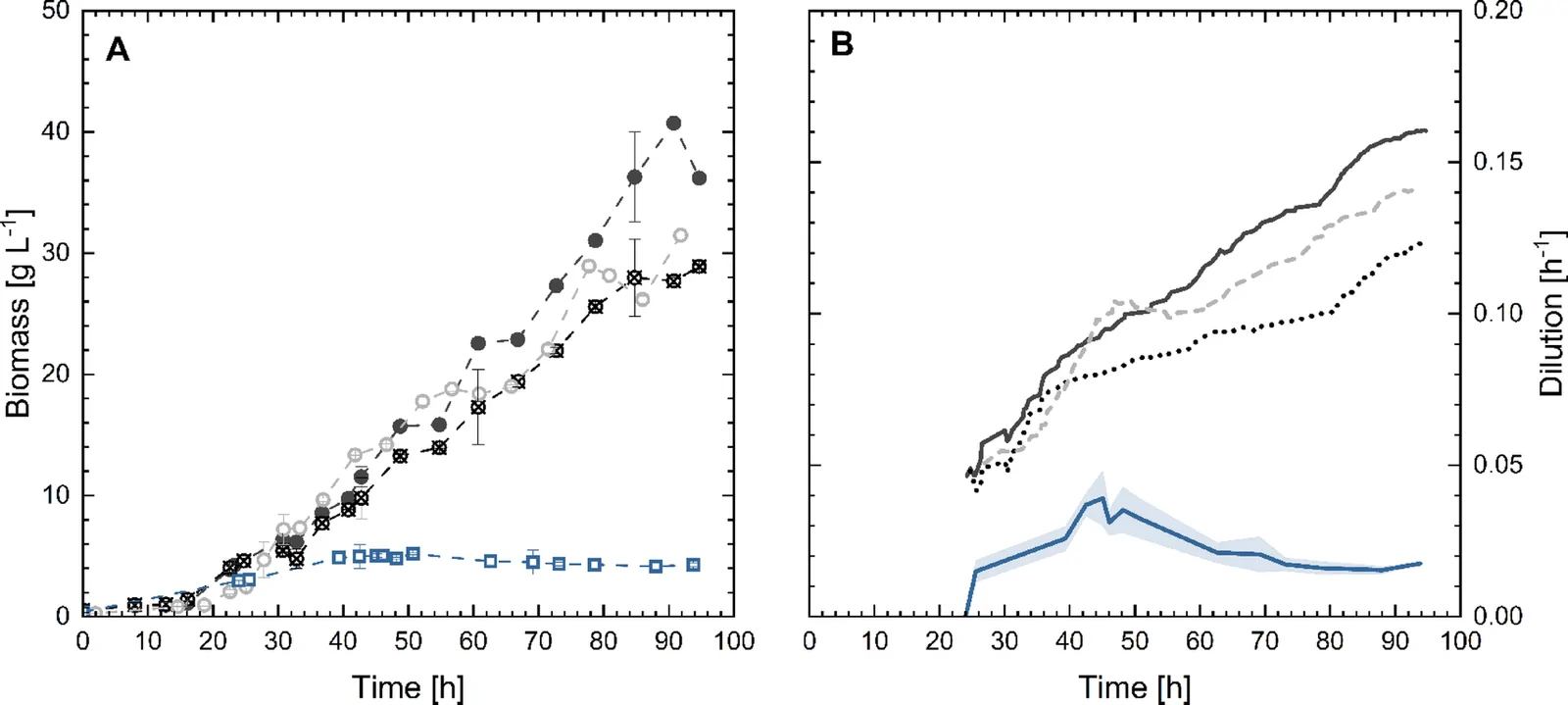
Biomass accumulation and dilution rate of S. limacinum SR21 cultures in repeated fed-batch and continuous operation mode with cell retention. **A**: Time course of biomass concentration for repeated fed-batch (blue squares) and cell retention cultivations CR1 (black filled circles), CR2 (black crosses), and CR3 (grey open circles); **B**: dilution rates, calculated from feed input relative to culture volume, for CR1 (black line), CR2 (black dashed line), CR3 (grey dashed line) and repeated fed-batch (blue line, events of volume reduction at 45 and 62 h). For CR, CDW values represent mean ± standard deviation from triplicate determinations, whereas dilution rates are shown as individual cultivation values. For RFB, CDW values represent the mean of duplicate measurements from independent cultivations, with error bars extending from the lower to the higher value. The RFB dilution rate is shown as the mean, with the shaded area extending from the lower to the higher value

The feed rate increased with biomass concentration, with no signs of decline observed during the 95-hour cultivation period. The average maximum feed rates were 156, 120, and 140 mL h⁻¹ for CR1 to CR3. The maximum dilution rates (F V^− 1^) in CR cultivation mode reached 0.12–0.18 h⁻¹, while they remained below 0.03 h⁻¹ in RFB cultivations (Fig. 3).

Together, these results show that repeated fed-batch was heavily constrained by medium withdrawal and dilution effects, which outweighed potential biomass gains and made it less suitable for feeds with low carbon concentrations such as DFE (Table 3). Similar dilution-related limitations were previously reported also for other substrates from residues (Chalima et al. 2019; Paul et al. 2019).

### SCCA uptake from DFE

The diauxic experiment indicated a clear preference for acetate and butyrate. Next SCCA uptake in CR cultivations was analysed. Concentration profiles and specific uptake rates were determined for acetate, butyrate, propionate, and lactate to assess which acids sustained growth, and to what extent DFE feeding aligned with experiments with defined SCCA mixtures (Table 2).

**Fig. 4:**
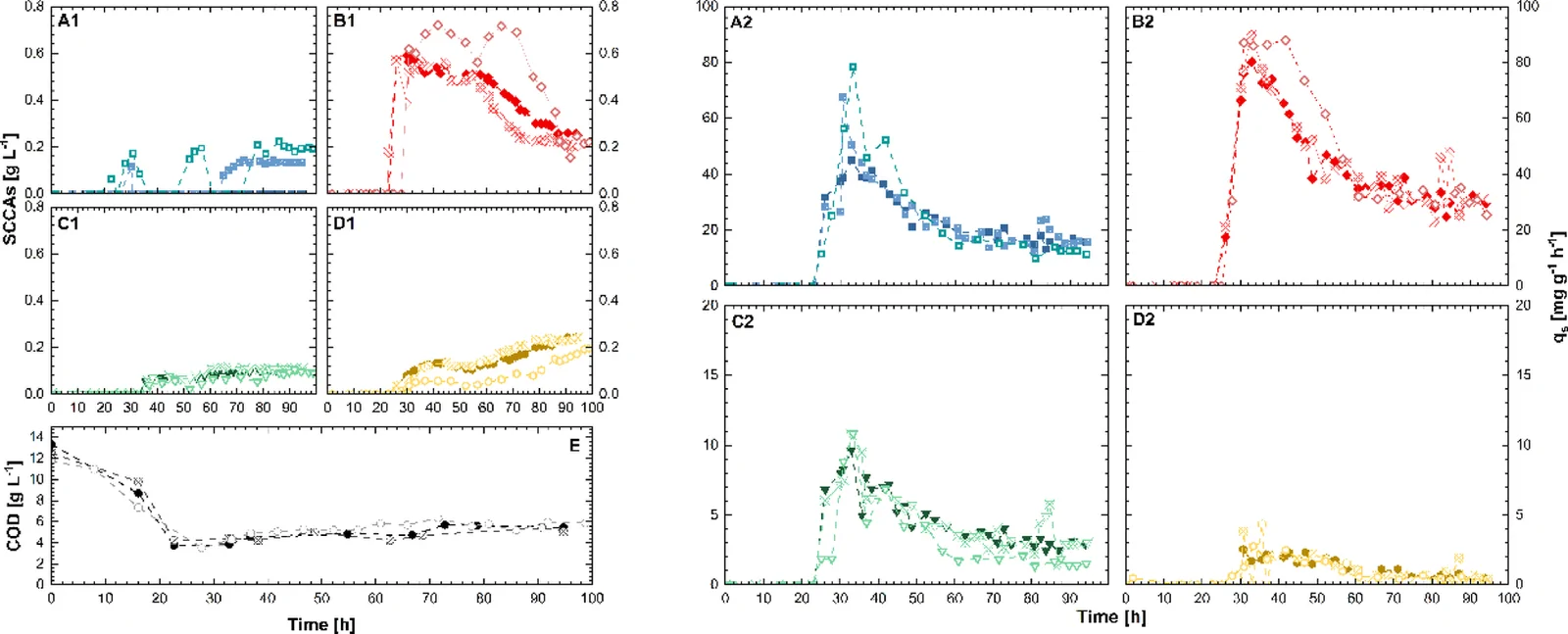
Performance of continuous *S. limacinum* cultivations with cell retention, fed with DFE. CR1 (filled symbols), CR2 (crossed symbols), and CR3 (open symbols). Time courses of acetate (**A1**), butyrate (**B1**), propionate (**C1**), and lactate (**D1**) concentration; E: chemical oxygen demand (COD); **A2**-**D2**: specific uptake rates (qₛ) for acetate (**A2**), butyrate (**B2**), propionate (**C2**), and lactate (**D2**). CR1 to CR3 represent individual bioreactor cultivations. Symbols represent the mean of duplicate measurements, with error bars extending from the lower to the higher analytical value

Acetate generally remained detectable at low levels. CR2 showed overall stronger growth leading to a complete uptake of acetate, while for CR3, a stable concentration of 0.14 g L⁻¹ was measured at 70 h. In CR1, higher initial acetate in the feed resulted in 0.2 g L⁻¹ at the end of the run. Concentrations often dropped below the detection limit, likely due to fluctuations in reactor filling. Overall, the uptake exceeded the feed supply in the early phase, reflected in an initial q_s_ peak that declined to a stable value of 20 mg g⁻¹ h⁻¹, closely matching the supply by feeding (Fig. 4).

Butyrate was also detectable, but the concentration declined steadily from 0.6 to 0.2 g L⁻¹. In CR1, the higher initial feed concentration delayed this decline. Uptake peaked at q_s,max_ = 94 mg g⁻¹ h⁻¹, but rates decreased over time, settling at an overall q_s_ of 46 mg g⁻¹ h⁻¹, which is twice as high as the uptake of acetate. This is consistent with its higher availability. Additionally, the decrease in uptake suggests that factors beyond substrate availability influence uptake rates. One possible explanation might be the limitation of other essential nutrients.

Propionate concentrations increased with the onset of feeding, reaching 0.1 g L⁻¹, before remaining stable. Specific uptake rates also decreased over time, reaching an overall q_s_ of 3 mg g⁻¹ h⁻¹.

The lactate concentration continuously increased, gradually approaching the average feed concentration of 0.25 g L⁻¹, indicating negligible uptake. The calculated apparent q_s_ remained below 0.5 mg g⁻¹ h⁻¹.

Compared to acetate and butyrate, uptake rates for propionate and lactate were lower. This suggests that cells continued to preferentially consume acetate and butyrate, while adaptation to less preferred SCCAs remained limited despite increasing propionate and lactate concentrations. This is consistent with the results when feeding defined SCCA mixture (Table 2). However, as cultivation progressed, carbon availability gradually shifted from preferred to previously less preferred sources. Rising propionate and lactate concentrations changed the acid balance.

Given the complex and variable composition of DFE, assessing both chemical oxygen demand (COD) and total molar carbon uptake provides a more comprehensive view of carbon assimilation and overall process performance (Fig. 4). COD remained stable at around 5,000 mg L⁻¹ throughout all CR runs. It is important to note that DFE contains additional COD sources beyond SCCAs. Specific uptake rates for carbon and COD during DFE feeding were, on average, 2.93 mmol_C_ g⁻¹ h⁻¹ and 107 mg_COD_ g⁻¹ h⁻¹.

In the RFB cultivation, the uptake hierarchy remained unchanged. Average q_s_ values over time (in mg g⁻¹ h⁻¹) were 24 (acetate), 34 (butyrate), 8 (propionate), or non-detectable (lactate). The maximum feed rate in RFB cultivations reached only 33 mL h⁻¹, compared to an average of 140 mL h⁻¹ in the CR cultivations. Specific carbon uptake was, on average, 2.65 mmol_C_ g⁻¹ h⁻¹, and the specific COD uptake rate was 107 mg_COD_ g⁻¹ h⁻¹, both comparable to CR cultivations. This supports the view that biomass-specific carbon uptake performance on DFE was similar across both modes, and that the performance gap mainly arose from dilution and washout in RFB cultivations.

**Table 2:** Specific uptake rates (q_s_) of short-chain carboxylic acids (SCCAs) during pH-auxostat cultivations of *S*. *limacinum* SR21.

Uptake rate	SCCA Mix	RFB	CR
q_s,max_ [mg g⁻¹ h⁻¹]			
Acetate	185 ± 8	51 ± 3	52 ± 1
Butyrate	163 ± 10	71 ± 6	94 ± 2
Propionate	78 ± 11	20 ± 3	9 ± 1
Lactate	13 ± 1	ND	ND
q_s_ [mg g⁻¹ h⁻¹]			
Acetate	44 ± 1	24 ± 1	20 ± 1
Butyrate	44 ± 1	34 ± 1	46 ± 3
Propionate	23 ± 1	8 ± 1	3 ± 1
Lactate	7 ± 1	ND	< 0.5
q_s,mol C_ [mmol_C_ g⁻¹ h⁻¹]	4.61 ± 0.08	2.65 ± 0.09	2.93 ± 0.13
q_s,COD_ [mg_COD_ g⁻¹ h⁻¹]	168 ± 8	107 ± 9	107 ± 11

Values for the defined SCCA mixture and RFB are reported as mean ± half range of measurements from two independent cultivations. CR values are reported as mean ± standard deviation across CR1 to CR3. Values represent maximum (qₛ,_max_) and average (qₛ) specific uptake rates of acetate, butyrate, propionate, and lactate for the SCCA mixture in repeated fed-batch (RFB) and cell retention (CR) bioreactor cultivations. ND = not detected. Specific carbon uptake in mmol_C_ g⁻¹ h⁻¹, specific COD uptake in mg_COD_ g⁻¹h⁻¹

### Lipid production

**Fig. 5:**
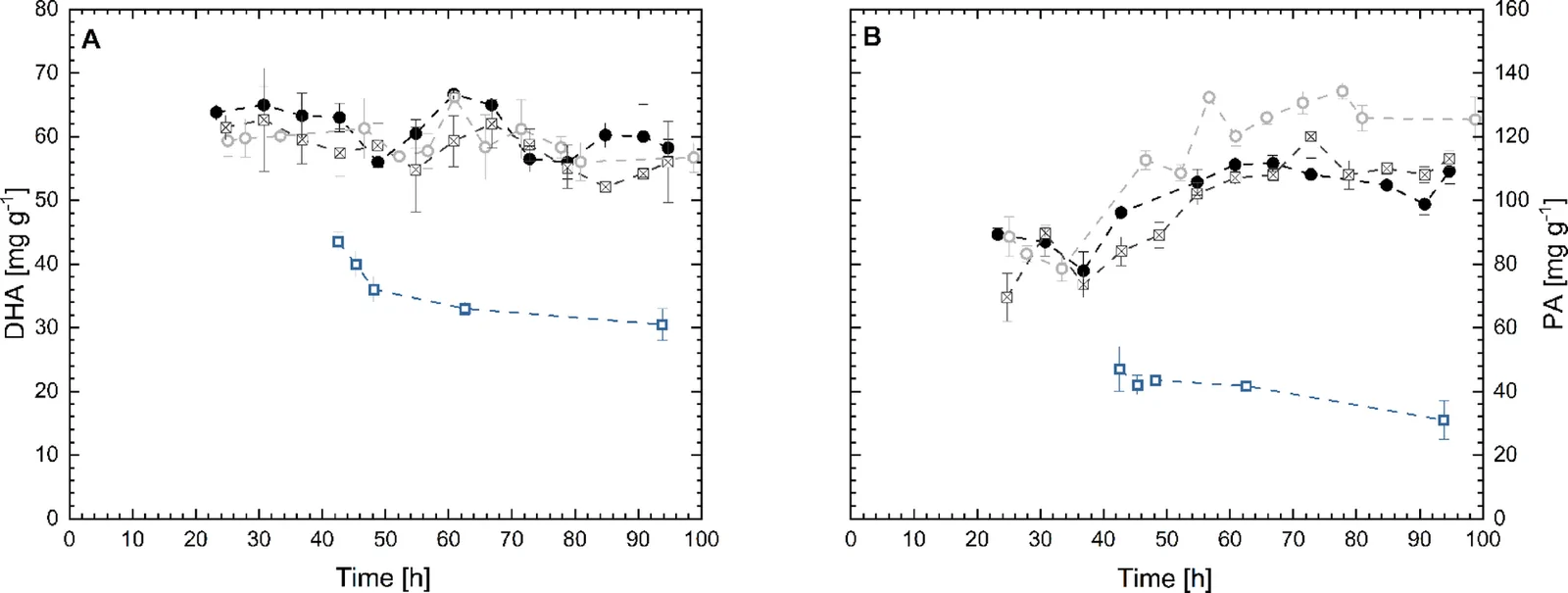
Lipid composition in *S. limacinum* SR21 cultures. Repeated fed-batch (RFB, blue squares) and cell retention cultivations: CR1 (filled circles), CR2 (crossed circles), and CR3 (open circles). **A:** Specific docosahexaenoic acid (DHA) concentration; **B:** specific palmitic acid concentration. Values represent mean ± standard deviation from at least triplicate measurements. RFB values represent the mean of duplicate measurements, with error bars extending from the lower to the higher value

The most abundant lipid products in *S. limacinum* are DHA and palmitic acid (PA). DHA is synthesised via the polyketide synthase (PKS) pathway, while PA originates from the fatty acid synthase (FAS) route (Tian et al. 2024; Liu et al. 2025). To evaluate DFE as a substrate for lipid production and its impact on both synthesis systems and cultivation modes, samples were taken every six hours and analysed by GC-FID (Fig. 5).

In CR cultivations, a clear shift toward PA accumulation was observed. PA increased from 89 ± 1, 70 ± 1, and 88 ± 3 mg g⁻¹ at 24 h to 109 ± 8, 113 ± 35, and 125 ± 15 mg g⁻¹ at 95 h for CR1-CR3, respectively. A maximum occurred between 70 and 80 h, coinciding with declining SCCA uptake rates and indicating a reduced carbon flux into the *de novo* lipid synthesis in the later phase. In contrast, the DHA content remained stable. It started at 63 ± 2, 61 ± 4, and 59 ± 4 mg g⁻¹ and declined only slightly to 58 ± 3, 56 ± 16, and 56 ± 5 mg g⁻¹ by 95 h. This suggests that the cell specific DHA concentration remained comparatively stable under DFE feeding, whereas PA accumulation increased during the later process phase. Combined with the measured uptake rates, this resulted in DHA yields of 591, 491, and 451 mg mol_C_⁻¹ and DHA titres of 2.25, 1.53, and 1.82 g L⁻¹ for CR1–CR3, respectively. A total lipid content of 18.7, 18.9, and 20.3% (w w^− 1^) was reached, leading to an overall titre of 6.8, 5.5, and 6.7 g L^− 1^, respectively.

The pattern was different in the RFB cultivation mode. DHA and PA both decreased continuously, reaching 31 ± 2 and 31 ± 6 mg g⁻¹ at 95 h. No lipid build-up occurred, resulting in a yield of 288 mg_DHA_ mol_C_⁻¹ and a cumulative DHA titre of only 0.24 g L⁻¹. A total lipid content of 6.9% and a titre of 0.52 g L^-1^ was achieved.

In summary, CR cultivations supported continued lipid accumulation, particularly of PA, while DHA remained comparatively stable. The resulting DHA yield and titre show that cell retention can overcome volumetric constraints by increasing biomass retention and overall lipid production. Although SCCA uptake and growth performance were similar between CR and RFB, lipid titres differed markedly. The higher lipid titre in CR was primarily due to greater retained biomass. The higher average intracellular lipid content in CR may also reflect prolonged biomass residence, which could allow part of the retained population to continue accumulating lipids over time.

### Nitrogen and phosphate

Nitrogen availability is a key regulator of lipid biosynthesis in *Schizochytrium* spp. When nitrogen is abundant, cellular resources are directed toward biomass formation. Under nitrogen limitation, growth slows down, and excess carbon is redirected into lipid accumulation. This metabolic switch is well established in oleaginous microorganisms and has also been demonstrated for *S. limacinum* (Heggeset et al. 2019; Liu et al. 2025). DFE typically contains nitrogen and phosphate. While this supports robust growth, it may delay the onset of nitrogen limitation and thereby restrict lipid induction. Phosphate, although essential for biomass formation, must also be removed from process streams to meet environmental discharge standards.

**Fig. 6:**
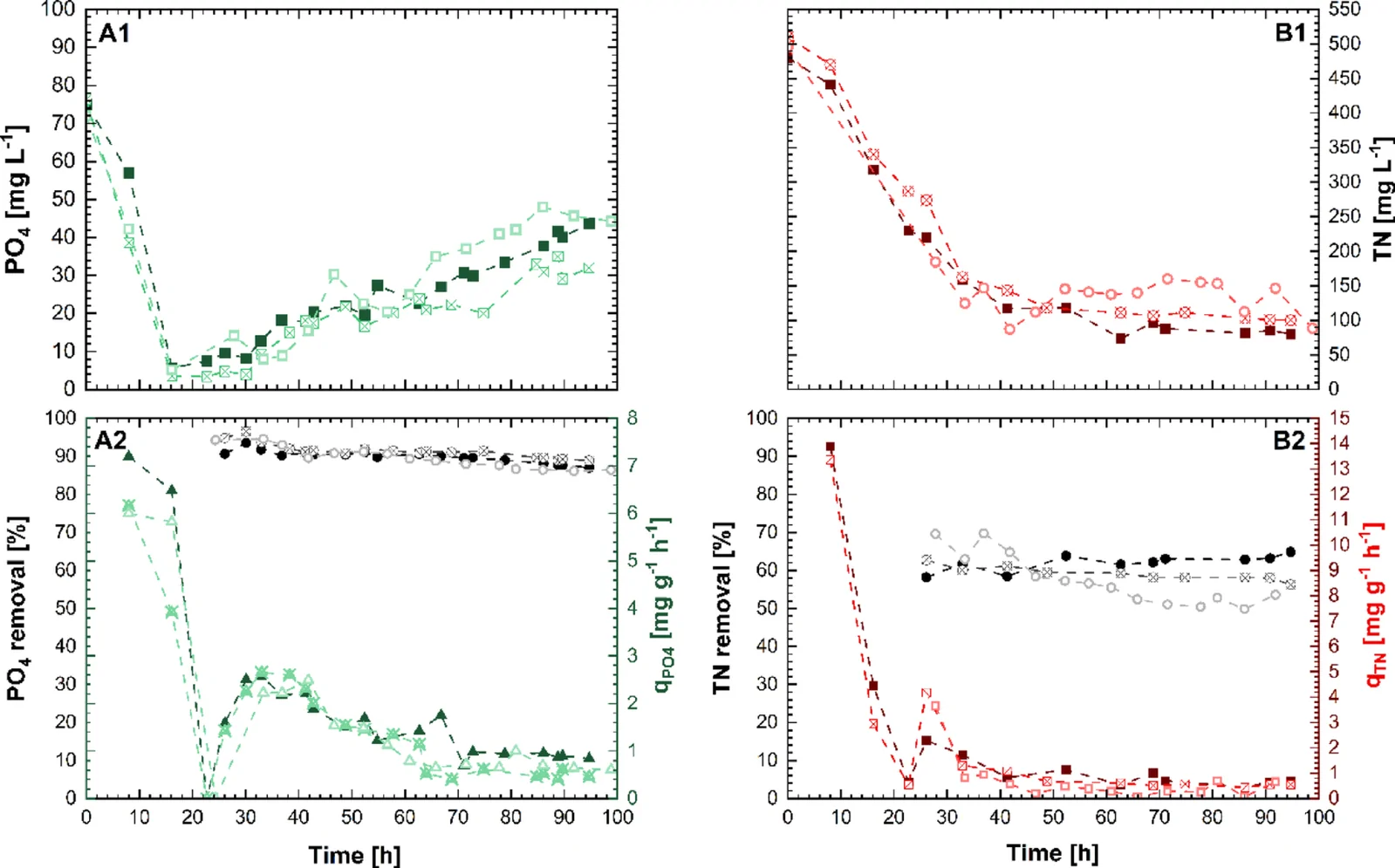
Nitrogen and phosphate removal in three cultivations of *S. limacinum* SR21 with DFE as feed and cell retention: CR1 (filled symbols), CR2 (crossed symbols), and CR3 (open symbols). **A1**: Phosphate (PO₄) concentrations; **B1**: Total nitrogen (TN) concentrations; **A2**: PO₄ removal and corresponding specific uptake rate q_S, PO₄_; **B2**: TN removal and specific uptake rate q_S, TN_. CR1 to CR3 represent individual bioreactor cultivations. Symbols represent the mean of duplicate measurements, with error bars extending from the lower to the higher value

To assess whether nutrient abundance interfered with assimilation and lipid accumulation, the total nitrogen (TN) and phosphate (PO₄) concentrations were measured during the process. PO₄ concentrations dropped to almost zero during the batch phase. Once DFE feeding began, PO₄ increased gradually. The uptake showed an early peak followed by a steady decline, reducing the removal efficiency from 95 ± 2% at 24 h to 87 ± 1% at 95 h (Fig. 6A). TN declined rapidly during the first 24 h, from 500 mg L⁻¹ to 220, 274, and 184 mg L⁻¹ in CR1-CR3, respectively. During DFE feeding, TN stabilised at 80–130 mg L⁻¹ (Fig. 6B). Specific nitrogen uptake rates peaked early and then dropped below 1 mg g⁻¹ h⁻¹, indicating rapid consumption of the readily assimilable nitrogen fraction. The applied DFE contained approximately 210 mg L⁻¹ TN, while nitrate and ammonia accounted for about 90 mg N L⁻¹ after conversion to nitrogen equivalents. This value closely matches the TN decrease observed during cultivation, suggesting that the remaining TN fraction was less readily bioavailable. The residual TN fraction should be interpreted with caution, because TN measurement does not distinguish between directly assimilable inorganic nitrogen and less defined organic nitrogen species.

**Table 3:** Key performance indicators of RFB and CR cultivation of *S. limacinum* SR21

Key performance indicators	RFB	CR
Cumulative biomass [g]	7.5 ± 0.9	32.7 ± 3.7
µ [h⁻¹], DFE feeding phase	0.031 ± 0.003	0.030 ± 0.005
Biomass productivity [g L⁻¹ h⁻¹]	0.14 ± 0.02	0.40 ± 0.05
Y_x/s_[g_CDW_mol_C_⁻¹]	10.5 ± 1.0	9.5 ± 0.6
DHA titre [g L⁻¹]	0.24 ± 0.07	1.87 ± 0.36
DHA yield [mg mol_C_⁻¹]	288 ± 21	511 ± 72
DHA STY [mg L⁻¹ h⁻¹]	2.5 ± 0.3	19.7 ± 3.8
Total lipid STY [g L⁻¹ h⁻¹]	0.005 ± 0.001	0.07 ± 0.01
TN removal [%]	76.0 ± 1.1	58.0 ± 3.9
PO₄ removal [%]	92.1 ± 0.2	87.0 ± 1.1
Total fed volume [L]	1.3 ± 0.2	6.9 ± 0.9
HRT [h]	–	10.4 ± 1.3

RFB values are reported as mean ± half range of measurements from two independent cultivations. CR values are reported as mean ± sample standard deviation across CR1 to CR3. Final biomass and lipid titre include the cumulative output for RFB. Specific growth rate (µ), biomass productivity, hydraulic retention time (HRT) were calculated for the DFE feeding phase (24–95 h). Biomass yield (Y_X/S_) refers to consumed molar carbon. DHA titre and yield are based on final measurements at 95 h for CR. DHA space–time yield (STY) was derived from the average titre and cultivation time. Total nitrogen (TN) and phosphate (PO₄) removal efficiencies represent the average relative assimilation from DFE. The biomass retention time in CR equals the process time

## Discussion

**Fig. 7:**
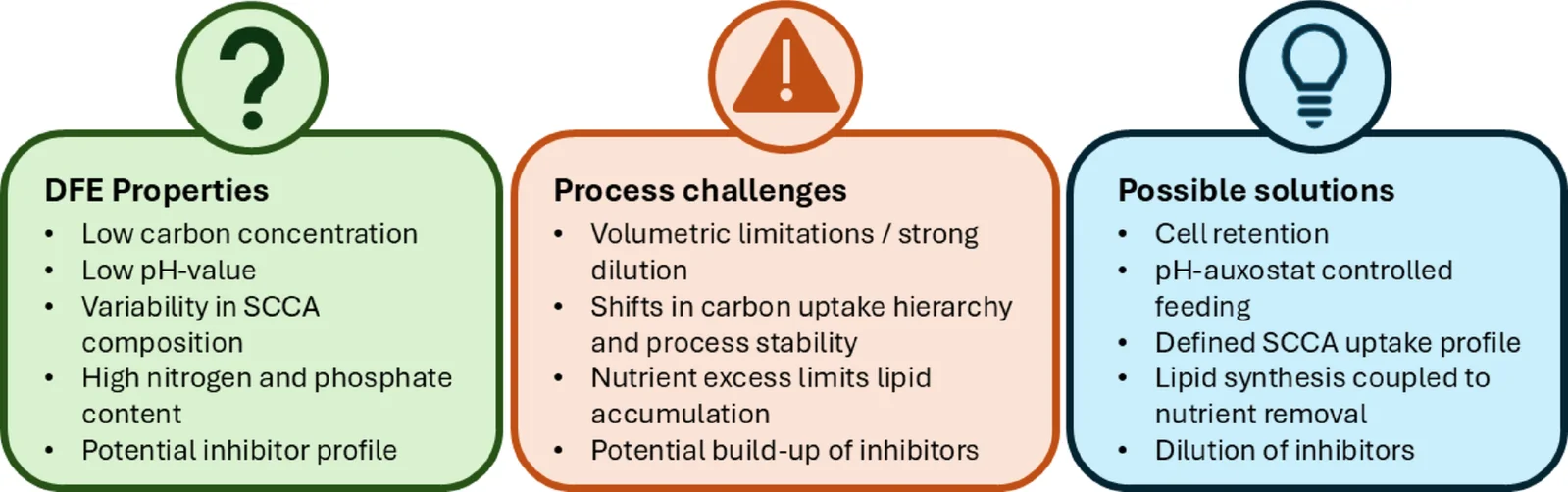
Operational window for DFE-based lipid production. Properties, challenges, and mitigation strategies for DFE as a feed for microbial lipid production. DFE is characterised by low carbon content, acidic pH, and variable composition, often containing excess nitrogen and phosphate and potentially inhibitory compounds. These traits introduce process challenges such as dilution, metabolic shifts, and inhibitor accumulation. These strategies help to manage dilution, substrate imbalance, and nutrient availability, thereby supporting volumetric lipid production under DFE feeding

Using DFE as feed poses inherent challenges and trade-offs that define its operational window (Täuber et al. 2025a). This study evaluates practical strategies to address some of these issues (Fig. 7).

First, the observed SCCA uptake hierarchy should be interpreted as a process constraint rather than as the mechanistic rationale for cell retention. Cell retention does not fundamentally change the substrate preference of *S. limacinum*. Instead, it increases the volumetric conversion capacity by retaining more biomass while allowing higher DFE throughput. Consequently, readily consumed acids such as butyrate and acetate were efficiently converted, whereas slowly consumed substrates, particularly lactate, accumulated when their feed rate exceeded the volumetric uptake capacity of the retained culture. Thus, DFE composition remains an important operational boundary for cell retention-based production.

This is particularly relevant because DFE typically has an acidic pH of 4–5 (Menzel et al. 2020), making it suitable for pH-auxostat feeding. In this mode, acid addition is linked to cellular SCCA consumption and the resulting increase in broth pH. However, the control signal depends on net acid consumption rather than on the uptake of each acid. Preferential uptake of acetate and butyrate increases the pH and triggers further DFE addition, while poorly consumed acids such as propionate and lactate can accumulate in the broth. As these acids accumulate, they reduce the net pH increase caused by SCCA consumption and thereby weaken the pH difference that drives feed addition. A decreasing or stagnating pH therefore does not necessarily indicate sufficient carbon supply but may also reflect the accumulation of poorly metabolised acids. Future operation should therefore combine pH-auxostat feeding with tighter feed composition control, base-supported pH correction, or a controlled pH decrease when the preferred acids become depleted. Based on the present results, three practical rules emerge for coupling DF with lipid production in *S. limacinum*: maintain high acetate and butyrate availability, restrict propionate accumulation, and minimise lactate formation in the upstream DF process.

The issue of low carbon concentration of DFE is addressed by cell retention. HFMs are especially valuable when feeding diluted waste streams, as discussed by Akkoyunlu et al. (2021). For example, 116 g L⁻¹ of biomass was achieved using yeast and a diluted feed containing 40 g L⁻¹ glucose and 10 g L⁻¹ mannose (Koruyucu et al. 2023). Also, other diluted feeds proved viable if cell retention was incorporated (Table 4). In this context, a further limitation can be membrane fouling during long-term operation or at high cell densities. HFMs can clog through pore blocking and cake formation, visible as rising transmembrane pressure and falling permeate flow. Stable operation requires fouling-aware set points, sufficient crossflow, and modules that delay blockage. Periodic backflushing and regular CIP/SIP are indispensable (Akkoyunlu et al. 2021). Although only one backflush was performed in the present study, higher biomass concentrations and longer cultivation times will likely increase fouling risks in future process development. However, higher cell density cultivation has been shown without severe membrane fouling for yeast up to 60 g L^− 1^ (Fu et al. 2018) and 116 g L^− 1^ (Koruyucu et al. 2023). In the present 95 h proof-of-concept cultivation, membrane performance was sufficient to maintain permeate withdrawal proving short-term operational feasibility. Nevertheless, the absence of transmembrane pressure monitoring limits the quantitative assessment of fouling and scalability.

Another benefit of cell retention is the continuous washout of soluble components which may limit the accumulation of potentially inhibitory compounds. DFE can contain chemically diverse inhibitory compounds, including furan derivatives, phenolic compounds, and organic acids such as formic and levulinic acid, depending on feedstock composition and upstream processing (Lyu et al. 2025). These compounds can impair growth in *Schizochytrium* and lipid production by disturbing membrane integrity, redox balance, and central metabolism (Wang et al. 2022). Individual inhibitors were not quantified in this study because the aim was to evaluate DFE performance at the process level rather than to resolve compound-specific toxicity.

However, the DFE used here originated from mechanically shredded lignocellulosic biomass and was produced under mild acidic, mesophilic, and anaerobic DF conditions without harsh chemical pretreatment (Longis et al. 2023; Menzel et al. 2023b). Such mildly hydrolytic conditions are expected to generate lower concentrations of furanic and phenolic inhibitors than acid catalysed pretreatments, as shown for sugar beet pulp hydrolysates by Cieciura-Włoch et al. (2019, 2024). Consistent with this, the present data did not indicate severe process-level inhibition by the applied DFE. *S. limacinum* maintained growth and metabolic activity during DFE feeding, as shown by continued SCCA uptake, biomass formation, nutrient assimilation, and lipid production. Although specific uptake rates of individual acids were lower during DFE feeding than in defined SCCA cultures, the average growth rate of approximately 0.03 h⁻¹ and the biomass yields were comparable to values obtained with defined SCCA media. Thus, inhibitors may remain relevant for DFE standardisation and scale up, but they were not the dominant limitation in the present process. The main constraints were the low carbon concentration of DFE, dilution effects, and the accumulation of slowly consumed acids such as lactate and propionate.

This leads to another important aspect: nitrogen and phosphate availability represent a central trade-off between nutrient assimilation and lipid production. As shown in Table 1, the applied DFE batches contained substantial amounts of TN and PO₄, with an average molar C/N/P ratio of approximately 36/1/0.2. This composition is comparable to typical growth phase media for *S. limacinum* with C/N ratios around 30/1 but remains far below the C/N ratios commonly applied for strong lipid induction, which range from approximately 60 to 150 (Dar et al. 2024). Thus, the nutrient profile of DFE was expected to support continued biomass formation and to limit the establishment of strong nitrogen limitation.

From an environmental perspective, the high assimilation rates observed for nitrogen and phosphate, particularly the substantial phosphate removal, highlight the potential of *S. limacinum*-based processes for nutrient assimilation from waste streams, as recently discussed by Contreras and Oviedo (2023). At the same time, this nutrient availability likely favoured biomass formation rather than maximal lipid induction per cell. The impact of nitrogen that is not available for assimilation by cells remains unclear, though. Heggeset et al. (2019) showed that nitrogen starvation strongly upregulates the FAS pathway, whereas PUFA synthase activity is only marginally affected, resulting in increased formation of FAS-derived fatty acids while DHA productivity remains comparatively stable. The increase in PA content during CR cultivation may therefore indicate mild nitrogen limitation in the later process phase, whereas the stable DHA concentration suggests that CR mainly improved volumetric DHA production through biomass retention rather than through stronger intracellular DHA induction.

This interpretation may also help explain why the 7.8-fold higher final DHA titre in CR exceeded the 4.4-fold increase in cumulative biomass output. At 95 h, the cell-specific DHA concentration was approximately 1.8-fold higher in CR than in RFB. This difference did not result from a pronounced increase in DHA during CR. Instead, DHA remained comparatively stable in the retained biomass, whereas it gradually declined during RFB cultivations. Cell retention may therefore have contributed to maintaining biomass with an established DHA content during the later cultivation phase. In RFB operation, periodic removal of culture broth reduced the residence time of previously formed cells and was followed by renewed biomass formation. If biomass formation proceeded faster than DHA synthesis, it might have reduced the specific DHA concentration. Additionally, the longer exposure to nutrient limitation in CR may have favoured maintaining the specific DHA concentration, although the individual contribution of nutrient availability and the different cell age distribution in both operation modes with respect to DHA accumulation cannot be fully resolved.

Compared to other lipid production processes employing cell retention, this study demonstrates both strengths and trade-offs (Table 4). Biomass concentrations of 32.7 g L⁻¹ and lipid contents of 19.3% are moderate in comparison to studies using sugar-based feeds. For example, Meo et al. (2017) reported 58 g L⁻¹ biomass with 53% lipid content, whereas Koruyucu et al. (2023) achieved higher values of up to 117 g L⁻¹ and 76% lipid under phosphate-limited conditions. The DHA titre of 1.9 g L⁻¹ is, however, comparably high for *Schizochytrium* cultures fed with SCCAs from biogenic residues or waste streams. Compared to the DFE-based process by Sathiyamoorthi et al. (2019), which reached 13.5 g L⁻¹ biomass and 2.7 g L⁻¹ lipids with *Cryptococcus albidus*, the present study achieved higher biomass concentrations and comparable specific lipid titres. In terms of lipid space time yield, 0.07 g L⁻¹ h⁻¹ was reached. This value is lower than those reported for carbohydrate-fed cultivations but remains notable given the lower carbon availability and higher complexity of DFE. Importantly, this study also reports near-complete assimilation of phosphate (87%) and a nitrogen removal of 58%, adding additional value to the process.

**Table 4:** Cell retention strategies for microbial lipid production. Overview of microbial lipid production processes using cell retention, hollow fibre membranes (HFM), and related membrane techniques

Organism	Substrate(s) [g L^− 1^]	Process conditions	Biomass [g L^− 1^]	Lipid content [w w^− 1^]; [g L^− 1^]	Yield [g g^− 1^]; productivity [g L^− 1^ h^− 1^]	Source
*Cutaneotricho-sporon oleaginosus*	Microalgae hydrolysate: 40.8 glucose, 1.95 mannose, 4.25 galactose 13.3 protein	Bioreactor, external HFM; Phosphate limitation; D = 0.065 h^− 1^; 30 °C;	58	53%; 30.6	Y_x/s_ 0.43 STY 0.33	Meo et al. (2017)
*Cutaneotricho-sporon oleaginosus*	40 glucose; 10 mannose	50 L bioreactor; external ceramic HFM; Phosphate limitation; D = 0.022 h^− 1^, 30 °C, pH 6.5	116	76.5%; 81.7	Y_X/S_ 0.32 STY 0.37	Koruyucu et al. (2023)
*Cryptococcus albidus*	75 glucose	1 L bioreactor; external HFM; C/*N* = 8; D = 0.36 h^− 1^, pH 6	49.2	46.3%; 22.7	Y_X/S_ 0.3 STY 0.41	Fu et al. (2018)
*Cryptococcus albidus*	Hydrolysed food waste: 4.8 reducing sugars	2 L bioreactor; internal ceramic filters; D = 0.01 h^− 1^, 30 °C, pH 5	13.5	20%; 2.7	Y_X/S_ 0.236	Sathiyamoorthi et al. (2019)
*Rhodococcus opacus*	Refinery wastewater: 4.5 (COD)	2.5 L bioreactor, external tubular ceramic membrane, D = 0.06 h^− 1^. pH 7, 30 °C	3.6	86%; 3.1	99% COD removal in 160 h	Paul et al. (2019)
*Yarrowia lipolytica* MUCL 28,849	52 glycerol	3 L bioreactor, submerged ultrafiltration membrane; C/*N* = 34, D = 0.006 h^− 1^	49.8	9.8%; 4.9	Y_X/S_ 0.67 Y_P/S_ 0.06	Tsirigka et al. (2025)
*Schizochytrium limacinum* SR21	Dark fermentation effluent: 24.6 (COD), 13.36 (SCCA), 0.563 mol L^− 1^ (carbon)	1 L bioreactor, external HFM, C/N/*P* = 35/1/0.2; D = 0.097 h^− 1^, pH 6.5, 30 °C	32.7	19.3% 6.3	Y_X/S_ 0.79, 0.375 gg_SCCA_^−1^ Y_P/S_ 0.13, 0.072 gg_SCCA_^−1^ STY 0.07	This study

## Conclusions

This study demonstrates that cell retention enables volumetrically intensified lipid production from low-concentration DFE with *S. limacinum* SR21. Cell retention enabled substantially higher DFE throughput, biomass retention, and volumetric DHA production than repeated fed-batch operation, without improving the intrinsic substrate conversion efficiency of cells.

A substrate hierarchy was identified, with acetate and butyrate as preferred carbon sources, propionate as tolerated, and lactate as the least suitable across experimental conditions. The moderate C/N ratios of the DFE supported biomass formation but limited lipid accumulation, highlighting the need for carbon supplementation or two-stage strategies to trigger stronger nitrogen limitation. From a bio-assimilation perspective, phosphate and nitrogen were efficiently removed, demonstrating that nutrient assimilation can be aligned with product formation.

Overall, the integration of pH-controlled feeding and cell retention provides a promising operational framework for DFE-based DHA/PUFA production. The present results demonstrate a proof-of-concept study. Longer cultivation times with quantitative monitoring of the membrane performance will enable a full evaluation of the long-term stability and a techno-economic assessment.

## Data Availability

The datasets used and analysed during the current study are available from the corresponding author on reasonable request.

## List of symbols

µ: Specific growth rate [h^−^^1^]
D: Dilution rate [h^−^^1^]
F: Feed [L h^−^^1^]
m: Mass [g]
q_s_: Specific substrate uptake rate [g g^−^^1^ h^−^^1^]
q_s,mol C_: Specific mol carbon uptake rate [mol_C_ g^−^^1^ h^−^^1^]
q_s,COD_: Specific uptake rate based on COD [g_COD_ g^−^^1^ h^−^^1^]
STY: Space-time yield [g L⁻^1^ h⁻^1^]
V: Liquid volume [L]
Y_P/S_: Substrate-specific product yield [g mol_C_^−^^1^]
Y_X/S_: Substrate-specific biomass yield [g mol_C_^-1^]

## Abbreviations

BRT: Biomass retention time
C/N/P: Carbon to nitrogen to phosphorus ratio
CDW: Cell dry weight
CIP: Cleaning in place
COD: Chemical oxygen demand
CR: Cell retention
DF: Dark fermentation
DFE: Dark fermentation effluent
DHA: Docosahexaenoic acid
EPA: Eicosapentaenoic acid
FAS: Fatty acid synthase
GC FID: Gas chromatography with flame ionization detection
HFM: Hollow fibre membrane
HPLC RID: High performance liquid chromatography with refractive index detection
HRT: Hydraulic retention time
ND: Non-detectable
PA: Palmitic acid
PKS: Polyketide synthase
PO₄: Phosphate
PUFA: Polyunsaturated fatty acid
RFB: Repeated fed-batch
RQ: Respiratory quotient
SCCA: Short-chain carboxylic acid
SIP: Sterilization in place
TN: Total nitrogen

## Declarations

### Use of generative AI tools

During the preparation of this work, the authors used ChatGPT (OpenAI), and DeepL Write (DeepL SE), to improve grammar, spelling, clarity, and readability. After using these tools, the authors reviewed and edited the content as needed and take full responsibility for the content of the publication.
